# Neoeriocitrin Targeting Beclin1 Deubiquitination and Autophagy in Osteogenic Differentiation of Human Dental Pulp Stem Cells

**DOI:** 10.1002/advs.202504378

**Published:** 2025-08-19

**Authors:** Yu Wu, Haotian Liu, Qunyi Wang, Ting Zhang, Rixin Chen, Qiao Yuan, Xin Tong, Wenrong Yang, Yin Xiao, Fuhua Yan

**Affiliations:** ^1^ Nanjing Stomatological Hospital Affiliated Hospital of Medical School Institute of Stomatology Nanjing University Nanjing 210008 China; ^2^ School of Life and Environmental Science Centre for Chemistry and Biotechnology Deakin University Geelong VIC 3217 Australia; ^3^ School of Medicine and Dentistry & Institute for Biomedicine and Glycomics Griffith University Gold Coast QLD 4222 Australia

**Keywords:** autophagy, beclin1, deubiquitination, human dental pulp stem cells, neoeriocitrin, osteogenic differentiation, thermal proteome profiling

## Abstract

Human dental pulp stem cells (hDPSCs) are dental‐derived mesenchymal stem cells with robust multipotent differentiation potentials, rendering them promising for bone tissue engineering. However, their differentiation relies on expensive, hard‐to‐control growth factors. Neoeriocitrin (Neo), a natural flavonoid, promotes cell proliferation and regulates alkaline phosphatase activities. However, Neo's effect on hDPSCs osteogenesis and bone regeneration is unknown. This study investigated Neo's impact on hDPSCs osteogenic differentiation and its mechanisms for bone regeneration. Neo effectively boosted hDPSCs osteogenic differentiation in vitro and facilitated bone regeneration in rat calvarial defects in vivo. Thermal proteome profiling revealed Neo directly binds Beclin1, validated by cellular thermal shift assay, molecular docking, and molecular dynamics. Neo stabilized Beclin1 by inhibiting ubiquitination‐mediated degradation, increasing autophagy in Neo‐treated hDPSCs. Furthermore, Neo‐enhanced osteogenic differentiation is activated by the Beclin1 network, pivotal for bone regeneration. Elucidating the Neo‐Beclin1 interaction provides insights into regulating hDPSCs differentiation and opens new avenues for enhancing bone regeneration strategies.

## Graphical abstract

Neoeriocitrin enhances osteogenesis in hDPSCs and bone regeneration by targeting Beclin1 to inhibit ubiquitination‐mediated degradation, thereby stabilizing Beclin1 and increasing its protein abundance to activate autophagy.

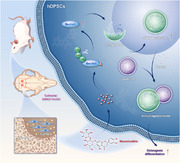

## 1 Introduction

Stem cells, with remarkable multipotent differentiation capabilities, have emerged as a compelling candidate in regenerative medicine.^[^ ^1^, ^2^ ^]^ Among these, dental pulp stem cells (DPSCs), a subpopulation of neural crest‐derived mesenchymal stem cells (MSCs), exhibit superior osteogenic differentiation potential,^[^ ^3^ ^]^ and unique advantages for clinical applications, including non‐invasive isolation (from discarded teeth), low immunogenicity, and the absence of ethical concerns, making them ideal seed cells for bone defect regeneration.^[^ ^4^, ^5^, ^6^, ^7^ ^]^ However, adult‐derived human dental pulp stem cells (hDPSCs) require exogenous stimulation to activate their osteogenic phenotype due to the lack of native microenvironmental cues.^[^ ^8^ ^]^ Conventional strategies, such as genetic modification or direct delivery of exogenous growth factors, are limited by challenges including uncontrollable gene therapy risks, high costs, adverse immune reactions, failed activities, and serious complications like ectopic bone formation and carcinogenesis, which severely restrict their clinical utility.^[^ ^9^, ^10^ ^]^ Thus, developing safe, stable, and cost‐effective new osteoinductive agents is pivotal to advancing hDPSCs‐based bone regeneration.

Recently, natural small‐molecule compounds, capable of mimicking microenvironmental signals to direct stem cell fate in a low‐cost, safe, and efficient manner, have garnered attention as a promising alternative for osteogenic induction.^[^ ^11^, ^12^ ^]^ Neoeriocitrin (Neo), a flavonoid compound extracted from *Drynaria fortunei* and certain citrus fruits, has demonstrated notable osteogenic potential. Studies showed that Neo upregulated key osteogenic markers (*Runx2*, *Collagen I*, and *OCN*) in MC3T3‐E1, counteracts PD98059 (an ERK inhibitor)‐mediated suppression of osteogenesis,^[^ ^13^ ^]^ and modulated bone remodeling by downregulating *SOST* and *RANKL* in osteocyte‐like cells.^[^ ^14^ ^]^ Despite these advances, critical gaps persist: 1) the osteoinductive effects of Neo on hDPSCs, a clinically superior cell source, remain unexplored; 2) mechanistic insights are confined to gene expression profiling, lacking in‐depth direct target identification; and 3) in vivo validation of Neo's bone regeneration efficacy is absent, hindering clinical translation. Notably, compared to potent osteoinductive cytokines with high clinical translation potential (e.g., BMP‐2, PDGF, FGF‐2), Neo, as a natural flavonoid, offers compelling advantages: its enhanced safety profile potentially circumvents BMP‐2‐associated risks of ectopic ossification and tumorigenesis,^[^ ^9^, ^10^ ^]^ significantly lower production costs from simplified plant extraction versus recombinant protein synthesis; and superior stability inherent to small‐molecule structures that bypass protein denaturation and cold‐chain constraints.

To leverage these advantages while addressing existing knowledge gaps, this study systematically investigated Neo's promoting effect on in vitro osteogenic differentiation of hDPSCs, and deciphered its precise target and molecular mechanism for the first time. Furthermore, we engineered a “Neo‐pre‐induced hDPSCs‐Bio‐Oss scaffold” composite, and evaluated its bone regeneration efficacy in a rat critical‐sized calvarial defect model. This study not only provides a natural compound‐driven approach for bone defect regeneration but also lays a theoretical foundation for the mechanistic research and translational application of plant‐derived active ingredients in stem cell therapy.

## 2 Experimental Section

### 2.1 Reagents

Alpha‐minimum essential medium (α‐MEM), fetal bovine serum (FBS), penicillin‐streptomycin, and tandem mass tag (TMT) were purchased from Thermo Fisher Scientific (Waltham, MA, USA). Neo was purchased from Push Bio‐technology company (HPLC ≥ 98.0%, Chengdu, China). RIPA lysis buffer, protease inhibitors, 1 × SDS‐PAGE loading buffer, and Bradford reagent were purchased from Beyotime (Shanghai, China). Bovine serum albumin (BSA) was purchased from NeoFroxx GmbH (Einhausen, Germany). β‐glycerophosphate, L‐ascorbic acid 2‐phosphate, dexamethasone, dithiothreitol (DTT), and hydroxylamine were purchased from Sigma‐Aldrich (St. Louis, MO, USA). 4% paraformaldehyde, phosphate buffer saline (PBS), and dimethyl sulfoxide (DMSO) were purchased from Biosharp (Hefei, China). Alizarin red solution was purchased from Oricell (Guangzhou, China). 2.5% glutaraldehyde was purchased from Servicebio (Wuhan, China). Cycloheximide (CHX) was purchased from Glpbio (Montclair, NJ, USA). Staurosporine (STS), 3‐Methyladenine (3‐MA), MG132, and chloroquine (CQ) were purchased from MedChemExpress (Monmouth Junction, NJ, USA). Pentobarbital sodium was purchased from Merck Millipore (Billerica, MA, USA).

### 2.2 Cell Isolation and Culture

In this study, hDPSCs were purchased from Lonza Bioscience (Lonza, Basel, Switzerland). The complete medium containing α‐MEM, 10% FBS, and 1% penicillin‐streptomycin was prepared and used to cultured hDPSCs under conditions of 5% CO_2_ at 37 °C. The cells were passaged every 2 or 3 days, and early‐passage (P2‐P5) hDPSCs were employed in the next set of experiments.

### 2.3 Cell Viability Assay

Cell proliferation and cytotoxicity were assessed using the cell counting kit‐8 (CCK‐8) (APExBIO, Houston, TX, USA). hDPSCs were plated in 96‐well plates at a density of 2 × 10^4^ cells mL^−1^ and cultured with varying concentrations of Neo (0, 2.5, 5, 10, 25, 50, 100, and 200 µM) for 1, 3, 5, 7, and 14 days. At each predetermined moment, the CCK‐8 reagent was added to the plates and incubated with the hDPSCs at 37 °C for 1 h, and the optical density (OD) values of the supernatant in the 96‐well plate were tested at a wavelength of 450 nm by a microplate reader (Molecular Devices, Sunnyvale, CA, USA).

### 2.4 Quantitative Polymerase Chain Reaction (qPCR)

Total RNA was extracted from the hDPSCs employing a cell/tissue total RNA isolation kit (Vazyme, Nanjing, China), cDNA was synthesized utilizing the HiScript III RT superMix for qPCR (Vazyme, Nanjing, China), and the osteogenesis‐related gene expression was subsequently detected by adding the ChamQ SYBR Color qPCR Master Mix (Vazyme, Nanjing, China). GAPDH served as an internal control, and the primers used are listed in Table S1 (Supporting Information).

### 2.5 Western Blotting

hDPSCs were lysed using RIPA lysis buffer containing 1% protease inhibitors to isolate the total proteins. Subsequently, 10 µg sample of denatured protein was exposed to electrophoresis using prefabricated gels and transferred onto 0.22 µm polyvinylidene fluoride (PVDF) membranes (Merck Millipore, Billerica, MA, USA). After being blocked with 5% BSA for 1 h, the membranes were incubated overnight at 4 °C with anti‐Collagen I (14695‐1‐AP, Proteintech, Wuhan, China), anti‐OPN (A21084, ABclonal, Wuhan, China), anti‐ALP (A0514, ABclonal, Wuhan, China), anti‐Runx2 (12556S, Cell Signaling Technology, Danvers, MA, USA), anti‐P62 (18420‐1‐AP, Proteintech, Wuhan, China), anti‐Beclin1 (11306‐1‐AP, Proteintech, Wuhan, China), anti‐LC3 (14600‐1‐AP, Proteintech, Wuhan, China) and anti‐GAPDH (M20050, Abmart, Shanghai, China), and then treated with goat anti‐mouse/rabbit secondary antibodies (BS12478/BS13278, Bioworld, Nanjing, China) for 1 h. Finally, the bands were observed with a chemiluminescence enhanced kit (ABclonal, Wuhan, China) and analyzed using the ImageJ software.

### 2.6 Alkaline Phosphatase (ALP) and Alizarin Red Staining (ARS)

When the cell density in the 12‐well plate, initially seeded at a density of 5 × 10^4^ cells well^−1^, reached ≈60%, the complete medium cultured hDPSCs was replaced with osteogenic differentiation medium, which comprised of α‐MEM, 10% FBS, 1% penicillin‐streptomycin, 10 mM β‐glycerophosphate, 50 µg mL^−1^ L‐ascorbic acid 2‐phosphate, and 0.1 nM dexamethasone. On day 7, the cells were treated with 4% paraformaldehyde for fixation and then stained with an ALP detection kit (Beyotime, Shanghai, China) to measure ALP expression. Similarly, on day 21, the hDPSCs were stained with alizarin red solution to evaluate mineralization.

### 2.7 Thermal Proteome Profiling (TPP)

TPP, in conjunction with a cellular thermal shift assay (CETSA) and quantitative mass spectrometry (MS),^[^ ^15^ ^]^ identified the direct target protein of Neo according to differences in the thermal stability of the ligand‐binding protein. hDPSCs were washed with PBS, digested with trypsin, and lysed through repeated freeze‐thaw cycles with liquid nitrogen. After quantifying the total protein using the Bradford reagent, the cell samples were diluted to a final concentration of 2 mg mL^−1^, transferred into two 1.5 mL eppendorf (EP) tubes, incubated with 100 µM Neo or an equal volume of DMSO for 1 h, and partitioned into 10 equal samples for 3 min heat treatment at different temperatures. The supernatant proteins were collected via centrifugation for subsequent assays. Further, 50 µg of the samples were placed in EP tubes, where 1% SDS and 10 mm DTT were introduced to react for 30 min. Subsequently, 25 mM iodoacetamide was added, and the cells were incubated for another 30 min, followed by the addition of 25 mM DTT for 15 min. Carboxyl magnetic beads for protein precipitation were then incorporated into the sample mixture, which was resuspended in EPPS, mixed at 37 °C, and digested with LysC and trypsin. After isolating beads and solution, the supernatants were TMT‐labeled and reacted at 1500 rpm for 1 h at 25 °C. The resulting mixtures were introduced into a hydroxylamine solution, stirred at 1500 rpm for 15 min, spin‐dried at 45 °C, fractionated using high‐performance liquid chromatography, and spin‐dried at 45 °C for subsequent MS analysis. KEGG (Kyoto Encyclopedia of Genes and Genomes)/Gene Ontology (GO) enrichment analysis was performed based on the top 10 Neo‐binding proteins identified by TPP (Table S3, Supporting Information). Fisher's exact test was implemented using the R package clusterProfiler (v4.0), with a significance threshold of *p*‐value ≤ 0.05 for enriched pathways/functions. The top 10 most significantly enriched GO terms/KEGG pathways were selected for visualization in bubble plots (Figure 2c,d).

### 2.8 CETSA

hDPSCs grown to 90% confluency in 100 mm dishes were incubated with either Neo or DMSO for 6 h, digested, and centrifuged at 1200 rpm for 5 min. The cells were resuspended in 300 µL PBS with protease inhibitors in the rearview window, aliquoted into 10 PCR tubes (30 µL each), and exposed to various temperature stimuli (37, 41, 44, 47, 50, 53, 56, 59, 63, and 67 °C) in a metal bath machine (Thermo Fisher Scientific, Waltham, MA, USA) for 4 min, followed by three cycles of freeze‐thawing in liquid nitrogen. Finally, the resulting supernatant was centrifuged at 16000 rpm for 20 min and detected using western blotting.

### 2.9 Molecular Docking

The structure of Beclin1 (PDB ID: 6HOJ) was downloaded from the Protein Data Bank (https://www.rcsb.org/) and preprocessed using PyMOL software to eliminate solvent and original ligands, whereas compound Neo (PubChem ID: 114627) was sourced from Traditional Chinese Medicine Systems Pharmacology Database and Analysis Platform (TCMSP, https://old.tcmsp‐e.com/tcmsp.php) and optimized using Open Babel software. AutoDock software was employed for conducting hydrogenation, dehydration, and molecular docking analyses. Eventually, the 3D structural diagram of the receptor‐ligand interactions was visualized using PyMOL software, whereas the corresponding 2D representation was generated using LigPlot software.

### 2.10 Molecular Dynamics Simulation

Initially, hydrogen atoms were added to the obtained ligand and protein structures using the YASARA software. The AMBER force field was selected to generate a topology file for the ligand Neo on the Acpype platform, whereas the topology file for the Beclin1 protein receptor was generated using GROMACS 2018.8. Next, a cubic simulation box was constructed with a minimum distance of 1.0 nm between the box boundaries and the protein in all XYZ directions to ensure adequate space for water molecules. The SPC model water molecules were introduced into the simulation box, accompanied by the appropriate sodium and chloride ions to balance the system charges. The system then underwent energy minimization with a maximum of 50000 steps and a tolerance of 10.0 kJ moL^−1^ considered, along with the equilibration under canonical (NVT) ensemble at 310 K and the isothermal‐isobaric (NPT) ensemble at 1 bar. Ultimately, molecular dynamics simulations were executed for a duration of 100 ns, followed by root mean square deviation (RMSD) calculations.

### 2.11 mRFP‐GFP‐LC3 Puncta Assay

To monitor cellular autophagic flux, hDPSCs were plated onto confocal dishes overnight, subsequently transfected with adenovirus (MOI = 5, Hanbio, Shanghai, China) for 24 h according to the manufacturer's protocol, and treated with or without Neo (5 µM) for 48 h. Thereafter, the cells were fixed in 4% paraformaldehyde for 30 min, and the green (green fluorescent protein, GFP) and red puncta (red fluorescent protein, RFP) were observed under a confocal microscope (Nikon, Tokyo, Japan).

### 2.12 Transmission Electron Microscopy (TEM)

hDPSCs were treated with or without 5 µM Neo for 48 h and scraped using cell scrapers. After overnight fixation at 4 °C with 2.5% glutaraldehyde, the cells were exposed to a 1% osmium tetroxide solution for 1–2 h, dehydrated using a graded ethanol series and acetone, then embedded and heated at 70 °C overnight. Ultra‐thin slices (70–90 nm) were prepared and stained with lead citrate and a solution of uranyl acetate saturated with 50% ethanol. Autophagosomes in the hDPSCs were visualized using TEM (HITACHI, Tokyo, Japan).

### 2.13 Apoptosis Assay

Apoptosis was assessed using Annexin V‐FITC/PI Apoptosis Detection Kit (Elabscience, Wuhan, China). After treatment (5 µM Neo for 48 h; 1 µM STS or STS+5 µM Neo for 6 h), cells were centrifuged (300 × g, 5 min), washed with PBS, and resuspended in 100 µL 1 × Annexin V Binding Buffer. Cell suspensions were stained with 2.5 µL Annexin V‐FITC and 2.5 µL PI (50 µg mL^−1^) using the manufacturer's optimized half‐volume protocol, vortexed gently, and incubated for 15–20 min at room temperature in the dark. Finally, 400 µL 1 × Binding Buffer was added, and samples were immediately analyzed by flow cytometry.

### 2.14 Lentiviral Transfection

Recombinant lentiviruses engineered for knockdown and overexpression of Beclin1 genes, along with the corresponding negative control lentiviruses, were procured from Hanbio (Hanbio, Shanghai, China). hDPSCs were seeded in 60 mm culture dishes at a density of 1.5 × 10^6^ cells dish^−1^ and incubated overnight. The cells were then transfected with the lentivirus (knockdown: MOI = 50, overexpression: MOI = 200) when they reached 60–70% confluence. The medium was refreshed 24 h post‐transfection, and the transfection efficacy was examined 48 h later.

### 2.15 Co‐Immunoprecipitation (Co‐IP)

Following the manufacturer's instructions, Co‐IP assays were conducted using a Co‐IP Kit (ACE Biotechnology, Changzhou, China). Briefly, hDPSCs were washed twice with PBS and lysed using 0.3% 1 × Lysis/Wash Buffer (Enhanced) containing protease inhibitors at 4 °C for 5 min. The cell supernatant was collected after centrifugation at 13000 × g for 10 min. For input controls, total lysates were quantified by BCA, and equal amounts were loaded to eliminate total protein differences. For immunoprecipitation, samples were incubated for 2 h with rProtein A/G MagPoly beads pre‐incubated with 5 µg IgG (B30011M, Abmart, Shanghai, China) or anti‐Beclin1 primary antibodies (3495S, Cell Signaling Technology, Danvers, MA, USA). All immunoprecipitation samples were washed thrice with 0.3% 1 × Lysis/Wash Buffer (Enhanced), denatured in 1 × SDS‐PAGE loading buffer, and subjected to analyzed by western blotting.

### 2.16 Animal Experiment

Twelve male Sprague‐Dawley (SD) rats aged 6 weeks, were selected for this study. All animal experiments were carried out at Nanjing Agricultural University and approved by the Laboratory Animal Welfare and Ethics Review Committee of Nanjing Agricultural University (No. PZW2023014). Following a 1‐week acclimatization period, a critical‐sized calvarial defect model was established. Pentobarbital sodium (1%, 40 mg kg^−1^) was administered intraperitoneally to anesthetize the rats according to their body weight. After fur removal, the surgical site was sterilized using iodine‐soaked cotton. An incision measuring ≈1.5–2 cm was made along the sagittal line of the skull, starting from the nasal bone. Layer‐by‐layer soft tissue dissection was performed to fully expose the sagittal suture, bilateral parietal bones, and portions of both frontal and occipital bones. Symmetrical drill holes were meticulously crafted on both sides of the sagittal suture utilizing a low‐speed handpiece and a trephine with an external diameter of 5 mm, operating at a drilling speed of less than 1500 rpm. Concurrent irrigation with sterile saline (1–2 drops sec^−1^) was employed to mitigate thermal injury. Twelve rats were allocated into four groups (3 rats per group, two symmetrical critical‐sized defects were created on each rat calvarium, yielding 6 defect samples per group for analysis), and bone substitutes were implanted into the bone defects as follows: 1) Blank, 2) Bio‐Oss (Geistlich, Wolhusen, Switzerland) (Bio‐Oss bone grafting materials alone), 3) hDPSCs/Bio‐Oss (hDPSCs mixed with Bio‐Oss bone grafting materials), and 4) Neo/hDPSCs/Bio‐Oss (P3 hDPSCs cultured with Neo for 5 days and then combined with Bio‐Oss bone grafting material at a seeding density of 1 × 10⁷ cells per 0.25 g of Bio‐Oss particles, which had a diameter range of 0.25–1.0 mm). Finally, an absorbable Bio‐Gide biofilm (Geistlich, Wolhusen, Switzerland) was used to cover the wound, after which the incision was sutured and disinfected. Penicillin was administered intraperitoneally for 3 days postoperatively. After 4 weeks, all rats were euthanized by anesthetic overdose, and skull and blood samples, along with major organs, were collected for further analysis.

### 2.17 Histological Staining and Immunohistochemistry (IHC)

Paraffin‐embedded specimens were cut into 4 µm sections, subsequently stained with hematoxylin and eosin (HE), Masson, and toluidine blue, and subjected to IHC analysis utilizing the avidin‐biotin‐peroxidase complex method with anti‐Beclin1 (11306‐1‐AP, Proteintech, Wuhan, China), anti‐P62 (A7758, ABclonal, Wuhan, China), anti‐LC3 (14600‐1‐AP, Proteintech, Wuhan, China) and anti‐Collagen I (ab270993, Abcam, Cambridge, UK) primary antibodies, in conjunction with the anti‐Mouse/Rabbit IHC secondary antibodies (MXB Biotechnologies, Fuzhou, China) and the DAB Plus Kit (MXB Biotechnologies, Fuzhou, China).

### 2.18 Statistical Analysis

The experimental data were analyzed using GraphPad Prism version 9.5, with results presented as the mean ± standard deviation (SD). Normality was assessed using the Shapiro‐Wilk test, and homogeneity of variances was evaluated with Levene's test. Comparisons between two groups used the unpaired Student's *t*‐test for normally distributed data with equal variances or the Mann‐Whitney *U* test for non‐normally distributed data or unequal variances. Comparisons among three or more groups used one‐way analysis of variance (ANOVA) followed by Tukey's post‐hoc test for pairwise comparisons when data conformed to both normality and homogeneity of variances, while non‐normally distributed data were analyzed using the Kruskal‐Wallis test followed by Dunn's test for post‐hoc pairwise comparisons. ^*^: *p* < 0.05, ^**^: *p* < 0.01, ^***^: *p* < 0.001.

## 3 Results

### 3.1 Neo Improved the Osteogenic Differentiation of hDPSCs in vitro

**Figure 1:**
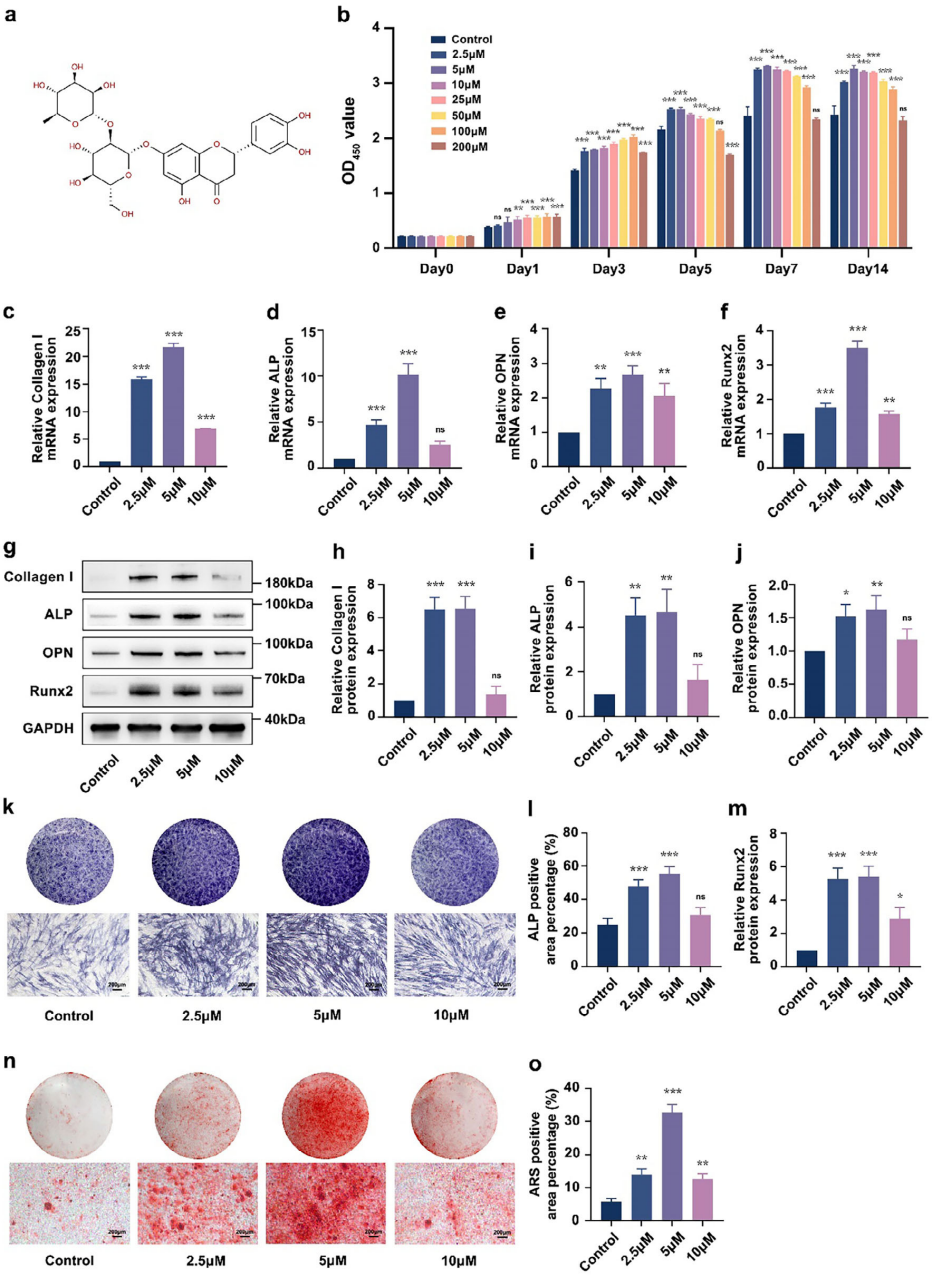
Neo improved the osteogenic differentiation of hDPSCs in vitro. a) The chemical structure of Neo. b) Proliferation of hDPSCs cultured in 0, 2.5, 5, 10, 25, 50, 100, and 200 µM Neo assessed by CCK‐8 assay. c–f) mRNA expression levels of *Collagen I*, *ALP*, *OPN*, and *Runx2* were measured by qPCR. g–j,m) Protein abundance of Collagen I, ALP, OPN, and Runx2 detected by western blotting. k,l) ALP staining of hDPSCs after 7 days of Neo treatment at various concentrations and the positive‐stained area analysis (Scale bar: 200 µm). n,o) ARS staining of hDPSCs after 21 days of Neo treatment at various concentrations and the positive‐stained area analysis (Scale bar: 200 µm). Data expressed as mean ± SD. ^*^: *p* < 0.05, ^**^: *p* < 0.01, ^***^: *p* < 0.001, ns: no significance.

The chemical structure of Neo is shown in **Figure** **1**a. Initially, the impact of varying Neo concentrations on the toxicity of hDPSCs was estimated using the CCK‐8 assay to identify the optimal concentration. Although the proliferation rate of hDPSCs generally increased with the augmentation of Neo concentrations except for 200 µM during the first 3 days, a conspicuous enhancement in proliferative capacity was observed in hDPSCs cultured with 2.5, 5, and 10 µM Neo compared with those cultured without Neo or with other concentrations of Neo from day 5 onwards (Figure 1b). Based on these results, 2.5, 5, and 10 µM Neo were selected for further experiments. As shown in Figure 1c–j,m, compared with 2.5 and 10 µM Neo, 5 µM Neo notably upregulated osteogenesis‐related mRNA and protein expression. Meanwhile, we observed that ALP and ARS staining after induction of mineralization for 7 and 21 days displayed trends consistent with the qPCR and western blotting findings (Figure 1k,l,n,o). Concurrently, these results indicate that Neo remarkably promotes the osteogenic differentiation of hDPSCs.

### 3.2 Neo Directly Targeted Beclin1 Protein in hDPSCs

**Figure 2:**
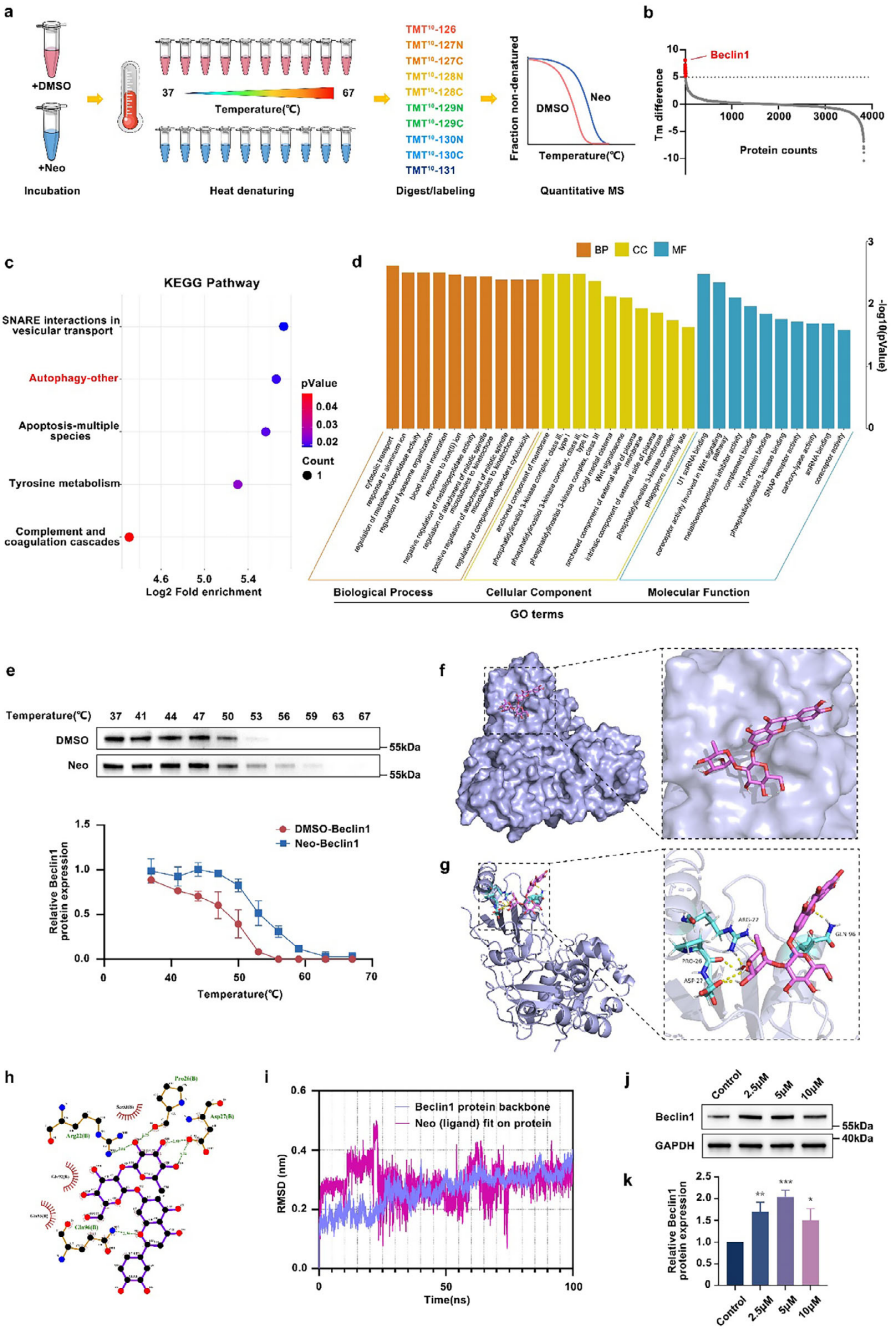
Neo directly targeted Beclin1 protein in hDPSCs. a) Schematic illustration of TPP. b) Distribution of the Tm difference of all proteins. The proteins with vital changes (Tm difference ≥ 5) are highlighted in red. c,d) KEGG and GO enrichment analyses of the regulatory pathways involving the top 10 proteins influenced by Neo in hDPSCs. e) CETSA is validating direct binding between Neo and Beclin1. f,g) 3D visualization of the interaction between Neo and Beclin1, with only the interacting residues labeled for clarity. h) The 2D image revealing Neo interacts with several amino acids. i) Molecular dynamics RMSD analysis. j,k) Beclin1 protein abundance detected by western blotting. Data expressed as mean ± SD. ^*^: *p* < 0.05, ^**^: *p* < 0.01, ^***^: *p* < 0.001.

TPP, a recently proposed label‐free technique for identifying drug targets, operates on the principle that proteins that are coupled to a ligand show more resistance to heat‐induced unfolding.^[^ ^16^, ^17^ ^]^ The Neo target proteins were found using this approach. A total of 6585 proteins were identified in hDPSCs, including 4858 quantifiable proteins (Table S2, Supporting Information), among which a total of 3826 proteins satisfied the analysis criteria for calculated half‐melting temperature (Tm) (R^2^ > 0.8;plateau < 0.3) in samples treated with 100 µM Neo, compared with those treated with DMSO (Table S3, Supporting Information). Only the top 10 proteins with a remarkable alteration in thermal stability (Tm difference ≥ 5) under Neo conditions are shown in **Figure** **2**b, of which Beclin1, which had the highest analytical value, was considered. Beclin1 is recognized as a key regulatory protein involved in autophagy, closely associated with bone formation. Moreover, subsequent KEGG and GO enrichment analyses indicated that the primary regulatory pathways influenced by Neo treatment in hDPSCs were closely related to autophagy (Figure 2c,d). Consequently, Beclin1 was selected as a prospective target of Neo for further validation.

To corroborate the above findings, we conducted CETSA and found that with increasing temperature, Beclin1 protein abundance exhibited observably reduced degradation following Neo treatment compared with DMSO treatment (Figure 2e), suggesting a strong interaction between Neo and Beclin1. Molecular docking analysis was performed to validate this interaction further. As illustrated in Figure 2h, Beclin1 had an active binding pocket conducive to Neo‐insertion. Neo binds to Beclin1 primarily through four hydrogen bonds at Arg22, Pro26, Asp27, and Gln96, with additional stability from three hydrophobic interactions at Ser53, Gly92, and Gln93. The Neo‐binding mode is shown in Figure 2f,g. Although molecular docking simulates potential instantaneous binding states, it may not accurately represent stable or rational configurations in a dynamic environment. Hence, molecular dynamics simulations were conducted to analyze the RMSD over a 100 ns timeframe to further explore the binding stability between Neo and Beclin1. As depicted in Figure 2i, the RMSD curve of the Beclin1 protein and small molecule complex fluctuated within a narrow range of ≈0.4 nm after 25 ns, lacking significant deviations, which indicates that the complex formed by the Beclin1 protein and the small molecule Neo possesses high stability. Concurrently, western blotting analysis confirmed that 5 µm Neo considerably enhanced the abundance of Beclin1 protein (Figure 2j,k). According to these results, Neo binds directly to Beclin1.

### 3.3 Neo Induced Osteogenic Differentiation by Activating Autophagy

**Figure 3:**
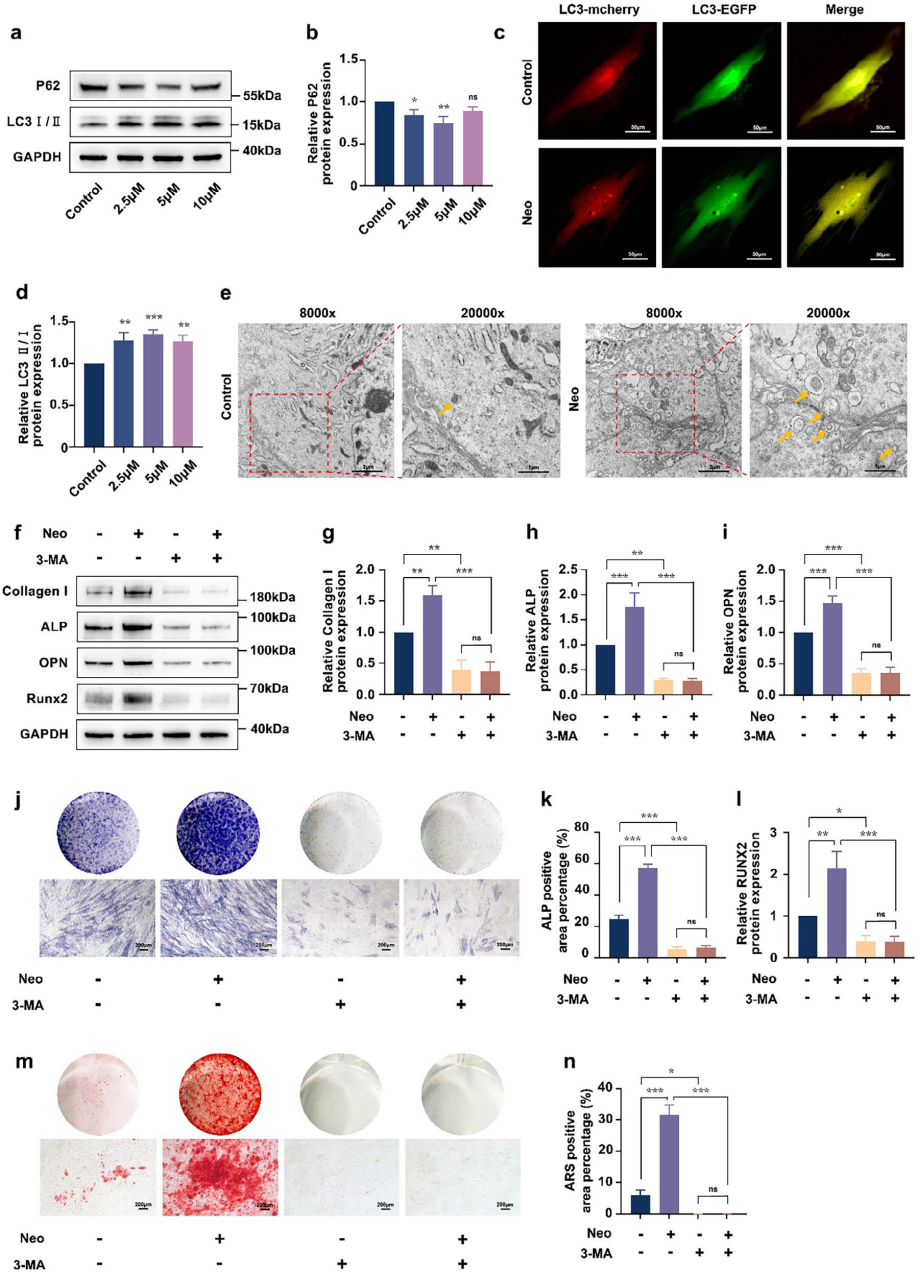
Neo induced osteogenic differentiation by activating autophagy. a,b,d) P62 and LC3 II/I protein abundance detected by western blotting. c) Representative fluorescence imaging photographs of hDPSCs transfected with mRFP‐GFP‐LC3 adenovirus and then incubated with or without Neo (5 µM) for 48 h (Scale bar: 50 µm). e) Representative pictures of TEM in hDPSCs treated with or without Neo (5 µM) for 48 h. Arrows show autophagosomes (Scale bar: Left, 2 µm; Right, 1 µm). f–i,l) Alterations in the protein abundance of Collagen I, ALP, OPN, and Runx2 were detected by western blotting following under 5 mM 3‐MA treatment with or without Neo treatment. j,k) ALP staining of hDPSCs was conducted at 7 days under 5 mM 3‐MA treatment with or without Neo, and the positive stained area analysis (Scale bar: 200 µm). (m, n) ARS staining of hDPSCs was conducted at 21 days under 5 mM 3‐MA treatment with or without Neo, and the positive‐stained area analysis (Scale bar: 200 µm). Data expressed as mean ± SD. ^*^: *p* < 0.05, ^**^: *p* < 0.01, ^***^: *p* < 0.001, ns: no significance.

To investigate whether Neo activates autophagy in hDPSCs, the protein abundance of autophagy‐related genes was detected using western blotting. Western blotting analysis revealed that treatment with 2.5 and 5 µm Neo significantly reduced P62 protein levels and elevated the LC3‐II/I ratio compared to control cells (**Figure** **3**a,b,d), demonstrating effective induction of autophagy at these concentrations. Notably, this reduction in P62 was concentration‐dependent, with 10 µM Neo showing a diminished and statistically non‐significant effect on P62 levels (Figure 3a,b). This result was further supported by the noteworthy increase of yellow puncta (autophagosomes, RFP^+^ GFP^+^) in Neo‐treated cells compared with control cells in the merged images (Figure 3c). Supporting this observation, Neo‐treated cells contained a greater number of autophagosomes than control cells, as displayed by TEM (Figure 3e). Critically, Annexin V/PI flow cytometry confirmed that 5 µM Neo alone did not induce apoptosis and even partially attenuated STS‐induced apoptosis (Figure S1, Supporting Information), effectively ruling out apoptosis as a contributor to the observed Neo‐induced osteogenesis effects. Next, we administered 3‐MA, an autophagic inhibitor, to further explore the role of autophagy in Neo‐induced osteoblast differentiation. The application of the inhibitor impeded Neo's osteoinductive effect (Figure 3f–n), demonstrating that Neo‐activation‐mediated autophagy is involved in Neo‐induced osteogenic differentiation.

### 3.4 Beclin1 is a Key Factor for Neo‐Triggered Autophagy and Osteoblast Differentiation in hDPSCs

**Figure 4:**
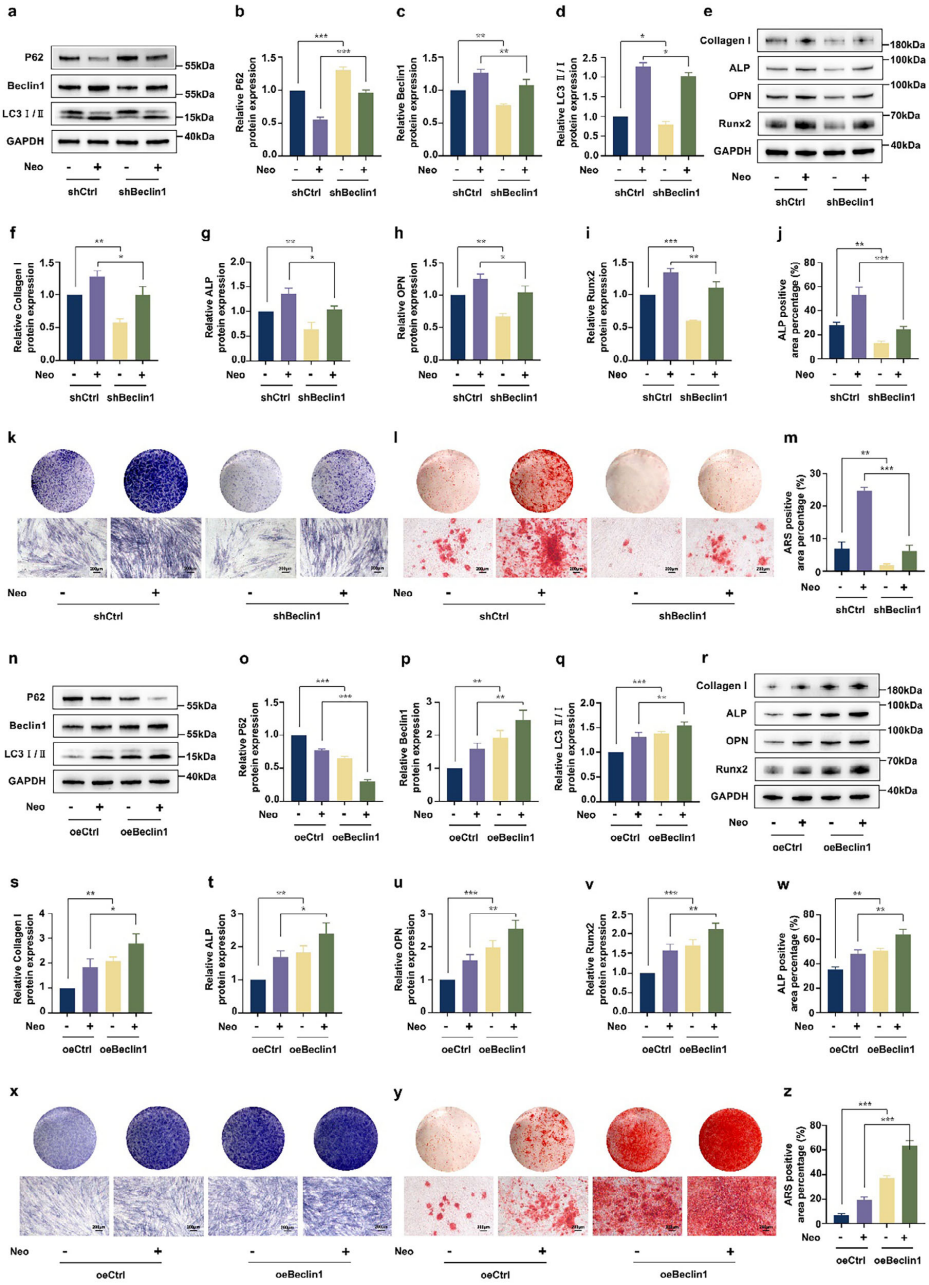
Beclin1 is a key factor for Neo‐triggered autophagy and osteoblast differentiation in hDPSCs. a–d) Alterations in the protein abundance of P62, Beclin1, and LC3 II/I were detected by western blotting following Beclin1 knockdown over time, with or without Neo treatment. e–i) Alterations in the protein abundance of Collagen I, ALP, OPN, and Runx2 were detected by western blotting following Beclin1 knockdown, with or without Neo treatment. j,k) ALP staining of hDPSCs was conducted at 7 days post‐Beclin1 knockdown, with or without Neo, and the positive stained area analysis (Scale bar: 200 µm). l,m) ARS staining of hDPSCs was conducted at 21 days post‐Beclin1 knockdown, with or without Neo, and the positive stained area analysis (Scale bar: 200 µm). n–q) Alterations in the protein abundance of P62, Beclin1, and LC3 II/I were detected by western blotting following Beclin1 overexpression, with or without Neo treatment. r–v) Alterations in the protein abundance of Collagen I, ALP, OPN, and Runx2 were detected by western blotting following Beclin1 overexpression, with or without Neo treatment. w,x) ALP staining of hDPSCs conducted at 7 days post‐Beclin1 overexpression, with or without Neo, and the positive‐stained area analysis (Scale bar: 200 µm). y,z) ARS staining of hDPSCs was conducted at 21 days post‐Beclin1 overexpression, with or without Neo, and the positive stained area analysis (Scale bar: 200 µm). Data expressed as mean ± SD. ^*^: *p* < 0.05, ^**^: *p* < 0.01, ^***^: *p* < 0.001.

To validate Beclin1 as a target of Neo's autophagic and osteogenic effects, we used lentiviral transfection to knock down and overexpressed Beclin1 in hDPSCs (**Figure** **4**a,c,n,p; Figure S2, Supporting Information), and rescue assays were designed to assess Neo's autophagy and osteogenic capacity in each scenario. These findings indicated that Neo was less capable of downregulating P62 and upregulating LC3 protein abundance in the shBeclin1 group than in the shCtrl group (Figure 4a,b,d). In the meantime, Beclin1 knockdown considerably impaired Neo's ability to increase the abundance of Collagen I, ALP, OPN, and Runx2 proteins (Figure 4e–i). The results of ALP and ARS staining were consistent with the findings of the western blotting analysis (Figure 4j–m). As a result, Beclin1 knockdown partially diminished the autophagic and osteogenic potential of Neo. In contrast, Neo treatment induced a significantly greater reduction in P62 protein abundance and a significantly higher increase in the LC3‐II/I ratio in the oeBeclin1 group compared to the oeCtrl group (Figure 4n,o,q). Furthermore, Beclin1 overexpression amplified the effects of Neo on the abundance of Collagen I, ALP, OPN, and Runx2 proteins (Figure 4r–v). This finding was corroborated by the ALP and ARS staining results, which aligned with the western blotting data (Figure 4w–z). Generally, these findings verify that Beclin1 is the key factor in modulating the autophagic and osteogenic potential of Neo.

### 3.5 Neo Stabilizes Beclin1 by Inhibiting Ubiquitin‐Mediated Degradation

**Figure 5:**
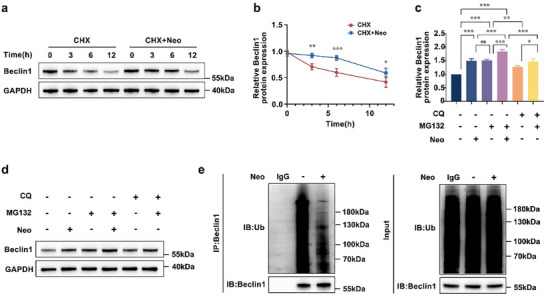
Neo stabilizes Beclin1 by inhibiting ubiquitin‐mediated degradation. a,b) Beclin1 protein abundance in hDPSCs was detected by western blotting following CHX (20 µg mL^−1^) treatment, in the absence and presence of Neo, over a specified duration. c,d) Beclin1 protein abundance in hDPSCs was measured by western blotting under conditions treated with either MG132 (10 µM) or CQ (10 µM), in the absence and presence of Neo. e) Input: Immunoblot of total lysates with equal protein loading. IP: Beclin1 immunoprecipitates probed with anti‐Ub antibody. Equal input loading eliminates total protein differences to focus on ubiquitination changes. Data expressed as mean ± SD. ^*^: *p* < 0.05, ^**^: *p* < 0.01, ^***^: *p* < 0.001, ns: no significance.

To define the mechanism of Neo‐mediated Beclin1 protein abundance, we first established that Neo treatment did not alter *BECN1* mRNA levels (Figure S3, Supporting Information), ruling out transcriptional regulation. We therefore hypothesized that Neo enhances Beclin1 stability by suppressing post‐translational degradation. CHX chase assays in hDPSCs confirmed this: Neo markedly delayed Beclin1 decay compared to CHX treatment alone (**Figure** **5**a,b), indicating prolonged half‐life. In eukaryotic cells, protein degradation mainly occurs through the ubiquitin‐proteasome pathway and the autophagy‐lysosome pathway.^[^ ^18^ ^]^ To identify the relevant degradation pathway, we combined Neo with lysosomal inhibitor CQ or proteasome inhibitor MG132. Critically, while CQ failed to dramatically enhance Neo‐induced Beclin1 abundance, MG132 synergistically amplified the effect (Figure 5c,d), specifically implicating the proteasomal degradation. Direct evidence emerged from Co‐IP: Under normalized protein input, Neo substantially reduced ubiquitin (Ub) conjugation to Beclin1 (Figure 5e)—despite increasing total Beclin1 (Figure 4c)—conclusively indicating attenuated ubiquitin‐mediated degradation. Collectively, Neo stabilizes Beclin1 protein abundance by selectively inhibiting its ubiquitination, thereby shielding it from proteasomal destruction and extending functional protein persistence in hDPSCs. This Neo‐mediated stabilization of Beclin1 provides the molecular basis for the observed increase in autophagy flux and consequent enhancement of osteogenic differentiation in hDPSCs (Figure 1 and Figures 3 and 4).

### 3.6 Transplantation of Neo‐Treated hDPSCs with Bio‐Oss Facilitated the Regeneration of Rat Calvarial Defect by Modulating Autophagy in vivo

**Figure 6:**
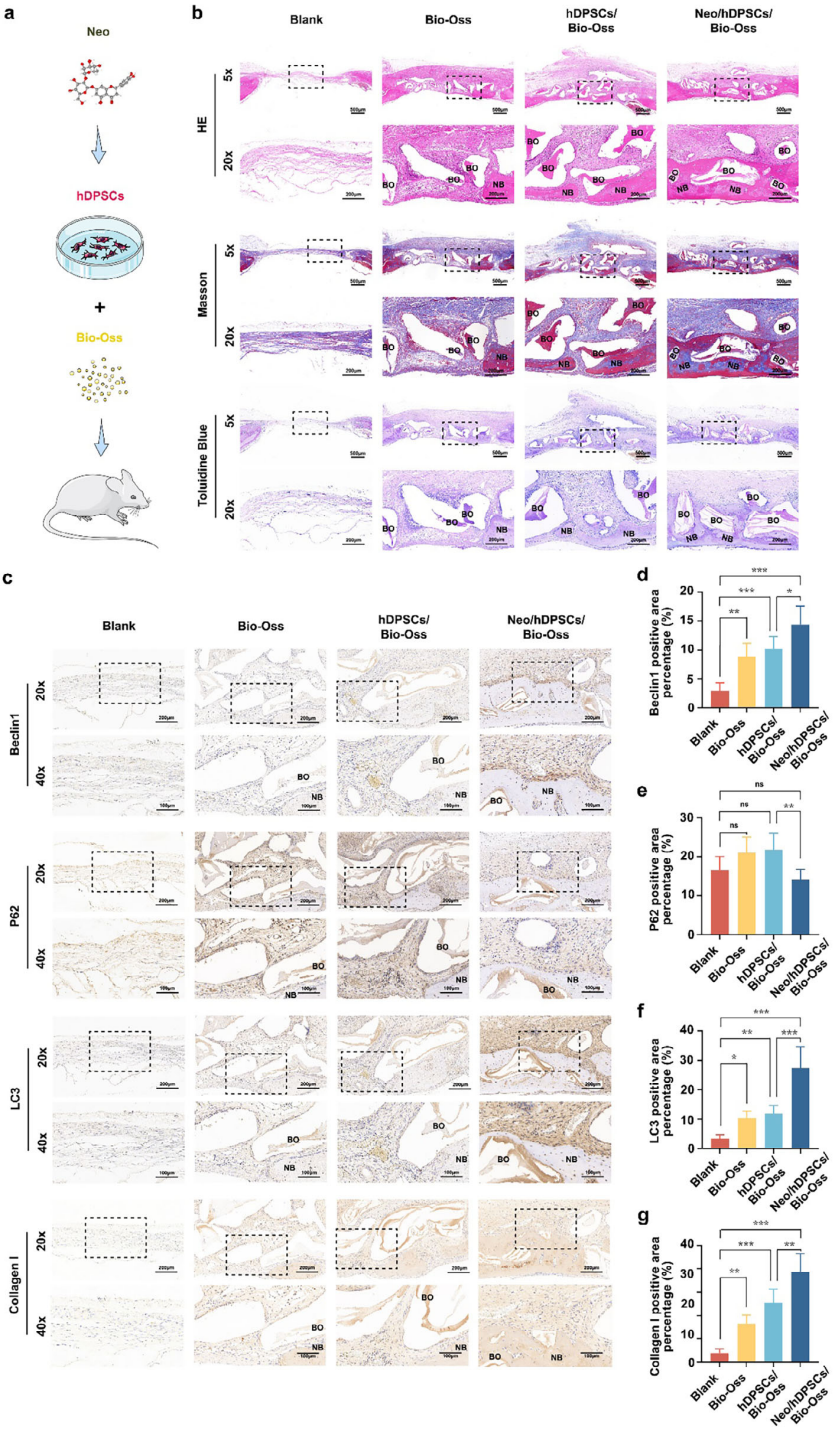
Transplantation of Neo‐treated hDPSCs with Bio‐Oss facilitated the regeneration of rat calvarial defect by modulating autophagy in vivo. a) The diagram depicting the procedure for transplanting Neo/hDPSCs/Bio‐Oss into rat calvarial defect regions. b) Representative images of HE, Masson, and toluidine blue staining of rat calvarial defect regions (NB: new bone; BO: Bio‐Oss) (Scale bar: Up, 500 µm; Down, 200 µm). c) Representative pictures of IHC staining for Beclin1, P62, LC3, and Collagen I in rat calvarial defect regions (Scale bar: Up, 200 µm; Down, 100 µm). d–g) The percentage of Beclin1, P62, LC3, and Collagen I positive area in different groups of rats. Data expressed as mean ± SD. ^*^: *p* < 0.05, ^**^: *p* < 0.01, ^***^: *p* < 0.001, ns: no significance.

To explore the influence of Neo on the osteogenic differentiation of hDPSCs in vivo and the potential translational application of our findings, the calvarial defect model was initially established in rats. The Bio‐Oss scaffold served as a carrier for hDPSCs and was implanted at the bone defect site. Bone tissue regeneration was evaluated 4 weeks after surgery. Histological analyses using HE, Masson, and toluidine blue staining revealed minimal new bone formation infiltrated by fibrous tissue in the Blank group. The Bio‐Oss group showed acellular Bio‐Oss particles surrounded by fibrous tissue, with limited new bone formation originating from the defect margin and sporadically around the scaffold. The hDPSCs/Bio‐Oss group demonstrated enhanced new bone formation around the Bio‐Oss particles and from the margins compared to Bio‐Oss alone. Strikingly, the Neo/hDPSCs/Bio‐Oss group exhibited the most substantial new bone formation, extensively covering the Bio‐Oss scaffold and occupying the largest proportion of the defect area (**Figure** **6**b). IHC analysis confirmed significantly elevated expression of Beclin1 and LC3, alongside reduced expression of the P62, within the defect sites of the Neo/hDPSCs/Bio‐Oss group compared to other groups (Figure 6c–f). Crucially, robust expression of Collagen I was specifically localized within the bone regeneration zones of the Neo/hDPSCs/Bio‐Oss group. These zones expressing Beclin1, P62, and LC3 exhibited precise spatial concordance with areas expressing Collagen I (Figure 6c). This colocalization pattern strongly suggests a functional coupling between enhanced autophagy and active osteogenesis at the transplantation site. Furthermore, the biosafety of Neo‐stimulated hDPSCs transplantation for bone defect treatment was evaluated using histological, blood‐routine, and blood biochemical analyses. HE staining of the hearts, livers, spleens, lungs, and kidneys of each group showed no obvious pathological alterations (Figure S4, Supporting Information). Compared with the Blank group, there were no notable statistical differences in blood‐routine and blood biochemical parameters between the other three groups (Tables S4 and S5, Supporting Information). These observations imply that the transplantation of Neo‐treated hDPSCs with Bio‐Oss positively affects bone regeneration. Neo contributes to bone regeneration in vivo by promoting the osteogenic differentiation of hDPSCs, potentially through the modulation of autophagy.

## 4 Discussion

The differentiation ability of stem cells is essential for bone tissue engineering. Various natural compounds, including hydroxysafflor yellow A,^[^ ^19^ ^]^ isonymphaeol B,^[^ ^20^ ^]^ hesperetin,^[^ ^21^ ^]^ and curcumin,^[^ ^22^ ^]^ are proven to offer promising avenues for enhancing MSCs and are thus considered effective strategies. This study is the first to report Neo augmenting osteogenic activity in hDPSCs and accelerating bone regeneration in rat calvarial defects through a novel mechanism where Neo directly targets Beclin1, stabilizes its protein abundance by inhibiting ubiquitination, and thereby activates autophagy to promote osteogenesis.

As shown in Figure 1b, while Neo at concentrations below 200 µm generally stimulated hDPSCs proliferation within the initial 3 days, this pro‐proliferative effect exhibited concentration‐dependency over time. Notably, higher concentrations (e.g., 50, 100 µM) showed diminished effects compared to the sustained enhancement observed with 2.5, 5, and 10 µM Neo from day 5 onwards. Crucially, further analysis revealed that the early proliferative response did not linearly predict the optimal osteogenic concentration. This was further evidenced by the concentration dependence observed in autophagy markers, which mirrored the osteogenic differentiation results. Specifically, 5 µM Neo emerged as the most potent concentration for enhancing osteogenic markers, mineralization activity (Figure 1c–o), and effective autophagy induction (as evidenced by significant P62 decrease at 2.5 and 5 µM and LC3‐II/I increase, Figure 3a,b,d). While 10 µM Neo showed comparable or slightly higher early proliferation (Figure 1b), it was significantly less effective than 5 µM Neo in both inducing osteogenesis (Figure 1c–o) and reducing P62 (Figure 3a,b). This concordance underscores that the optimal osteogenic effect of Neo at 5 µM is closely associated with its capacity to robustly activate autophagy at this specific concentration. The lack of significant P62 reduction at 10 µM Neo further reinforces this concentration‐dependent effect and correlates with its suboptimal osteoinductive effect observed at this higher concentration (Figure 1).

Although Neo facilitates osteoblast differentiation in MC3T3‐E1 cells and osteocyte‐like cells,^[^ ^13^, ^14^ ^]^ our study revealed that Neo significantly improved the osteogenic differentiation of hDPSCs in a dose‐dependent manner (Figure 1); however, the precise molecular mechanism responsible for this effect, particularly the basis for the superior efficacy of 5 µM Neo, is yet to be identified. To explore the underlying mechanism, we employed TPP to ascertain Neo's target information. Through the application of a matching methodology for osteogenesis‐related targets, we selected Beclin1 as the direct target of Neo and confirmed the binding between the receptor protein Beclin1 and the ligand Neo using CETSA, molecular docking, and molecular dynamics simulation (Figure 2a–i). Beclin1, a pivotal regulatory molecule in autophagy, plays a significant role in initiating autophagy and contributes to the formation of autophagosomes by interacting with various autophagy‐related factors, such as VPS34 and ATG14.^[^ ^23^, ^24^ ^]^ Autophagy is an essential intracellular degradation process that maintains the self‐renewal capacity and multipotent differentiation potential of stem cells by eliminating excess cellular components during osteogenesis.^[^ ^25^ ^]^ The activation of the pro‐autophagic AMPK‐BECLIN1 pathway has been reported to promote the osteogenic differentiation of stem cells.^[^ ^26^ ^]^ Furthermore, the activity of Beclin1 is intricately associated with signaling pathways (such as the Smad signaling pathway) that are pivotal to bone formation.^[^ ^27^ ^]^ As a result, elevating Beclin1 protein abundance to enhance stem cell‐mediated osteogenic differentiation is a promising strategy for facilitating bone regeneration based on stem cell therapy. However, there are currently no clinical drugs specifically targeting Beclin1; most research efforts have concentrated on preclinical studies of Beclin1 agonists. Recent studies indicate mTOR‐dependent osteogenic compounds (e.g., Morroniside) require overcoming mTOR‐mediated suppression of Beclin1/Atg13‐dependent autophagy.^[^ ^28^ ^]^ In contrast, Neo directly stabilizes Beclin1 by inhibiting ubiquitination (Figure 5), independent of mTOR signaling. This unique mTOR‐independent strategy circumvents osteogenesis inhibition risks from mTOR overactivation, offering a precise autophagy‐initiation targeting approach for bone regeneration. In this study, we found that Neo promoted Beclin1 protein abundance in hDPSCs (Figure 2j,k) while increasing the cells’ osteogenic differentiation by activating autophagy (Figure 3), which is consistent with previous studies emphasizing the significance of autophagy in bone formation.^[^ ^29^ ^]^ The observed increase in autophagosome formation (Figure 3c–e) carries significant functional implications. First, it substantially improves cellular quality control by selectively removing damaged organelles (e.g., mitochondrial fragments), thereby maintaining the intracellular homeostasis essential for osteogenic differentiation.^[^ ^30^, ^31^, ^32^ ^]^ Second, autophagy‐mediated metabolite recycling provides crucial energy support for this energy‐intensive process.^[^ ^33^, ^34^ ^]^ Therefore, the Neo‐driven enhancement of autophagy directly explains the significantly increased mineralization nodules observed in the Neo‐treated groups (particularly at 5 µm; Figure 1n,o and Figure 3m,n), as mineralization critically depends on ATP hydrolysis for energy.^[^ ^35^ ^]^ Concurrently, Annexin V/PI flow cytometry analysis confirmed 5 µM Neo neither induced apoptosis nor compromised viability, instead attenuating STS‐induced apoptosis (Figure S1, Supporting Information), definitively excluding apoptosis as a driver of Neo's osteogenic enhancement.

By knocking down and overexpressing Beclin1, we found that Neo correspondingly reduced and enhanced the differentiation capacity of hDPSCs (Figure 4), indicating that not only is Beclin1 crucial for Neo‐induced autophagic activation, but it also represents an important target for Neo‐mediated osteogenesis. Thus, targeting Beclin1 to promote autophagy offers a promising new strategy for bone regeneration. This study is the first to identify the natural compound Neo as a specific agonist of Beclin1. The significance of this discovery lies in its potential advantages for therapeutic development: 1) It overcomes the limitations of traditional growth factors, such as high cost and poor stability; 2) It avoids the risks associated with genetic therapies; 3) It establishes a foundation for developing dual‐function regulators targeting both “autophagy‐osteogenesis”. Based on this mechanism, Neo could serve as a novel stem cell priming agent to enhance the efficacy of stem cell‐based therapies, thereby advancing the clinical translation of bone tissue engineering approaches.

Through the knocking down and overexpression of Beclin1, we found that Neo correspondingly reduced and enhanced the differentiation capacity of hDPSCs (Figure 4), indicating that not only is Beclin1 crucial for Neo‐induced autophagic activation, but it also represents an important target for Neo‐mediated osteogenesis. Thus, targeting Beclin1 to promote autophagy offers a promising new strategy for bone regeneration. This study is the first to identify the natural compound Neo as a specific agonist of Beclin1. The significance of this discovery lies in its potential advantages for therapeutic development: 1) It overcomes the limitations of traditional growth factors, such as high cost and poor stability; 2) It avoids the risks associated with genetic therapies; 3) It establishes a foundation for developing dual‐function regulators targeting both “autophagy‐osteogenesis”. Based on this mechanism, Neo could serve as a novel stem cell priming agent to enhance the efficacy of stem cell‐based therapies, thereby advancing the clinical translation of bone tissue engineering approaches.

Moreover, maintaining the stability of Beclin1 is vital for autophagy, with ubiquitination identified as a key regulatory mechanism of Beclin1 stability.^[^ ^36^ ^]^ Typically, ubiquitination usually labels Beclin1 for degradation and inhibits its function in autophagy,^[^ ^37^, ^38^ ^]^ whereas deubiquitination can reverse this effect. For instance, the deubiquitinase ATAXIN3 can regulate Beclin1 stability during starvation‐induced autophagy, and the depletion of ATAXIN3 results in a reduction in Beclin1 levels and a concomitant decrease in autophagic flux.^[^ ^39^ ^]^ Two deubiquitinases, USP10 and USP13, may target Beclin1, and their interaction with Beclin1 ensures the stability of both Beclin1 and the deubiquitinases.^[^ ^40^, ^41^ ^]^ Our initial investigation ruled out transcriptional regulation, as Neo did not alter *BECN1* mRNA levels (Figure S3, Supporting Information). This prompted us to explore post‐translational mechanisms. Subsequent mechanistic dissection revealed that Neo stabilized Beclin1 by directly suppressing its ubiquitination (Figure 5e), synergized with proteasome inhibition (MG132) but not lysosomal blockade (CQ) (Figure 5c,d), and extended its half‐life (Figure 5a,b). This post‐translational stabilization increased Beclin1 protein abundance, thereby activating autophagy and boosting osteogenic differentiation of hDPSCs. These findings not only reinforce the established connections between autophagy and bone development, but also provide a novel theoretical foundation for Neo‐based bone repair strategies.

In addition to demonstrating that Neo enhanced the osteogenic activity of hDPSCs in vitro, we validated the bone repair potential of Neo using a rat calvarial defect model. The use of Bio‐Oss scaffold provided structural support, facilitating the retention and function of the transplanted stem cells within the defect site.^[^ ^42^ ^]^ Transplantation of hDPSCs preconditioned with Neo (followed by rigorous washing to minimize residual compound) significantly enhanced bone regeneration compared to untreated hDPSCs loaded onto Bio‐Oss scaffolds (Figure 6b). IHC analysis revealed robust Collagen I expression specifically localized within the defect area treated with Neo‐preconditioned hDPSCs (Figure 6c), confirming active osteoblast‐mediated bone formation. Critically, these regions also exhibited increased Beclin1 and LC3 expression alongside significantly reduced P62 levels (Figure 6c—f), demonstrating enhanced autophagy in vivo. The precise spatial concordance between the expression of these autophagy markers (Beclin1, LC3, P62) and Collagen I strongly supports functional coupling between Neo‐induced autophagy activation and osteogenesis within the regenerating tissue. This discovery lays novel scientific groundwork for the potential application of Neo in bone repair and fills existing gaps in prior in vivo studies.

Although our study yielded significant findings, it is important to acknowledge some limitations that require further investigation. Primarily, while the 4‐week endpoint effectively captured early osteogenic responses (Figure 6), future work should extend the observation period to 8–12 weeks to assess Neo‐treated hDPSCs' effects on long‐term bone maturation. Second, while spatial correlation strongly implicates the transplanted cells, definitive cellular attribution of Beclin1 expression within the regenerating niche requires future lineage tracing studies (e.g., using GFP‐labeled hDPSCs). Third, the therapeutic efficacy of transplanting Neo‐treated hDPSCs must be assessed in larger animal models, such as canines, before considering their clinical applications. Fourth, it remains unclear whether the interaction between Neo and Beclin1 modulates other downstream autophagy‐related proteins or whether it co‐regulates bone repair in conjunction with other signaling pathways, which requires further exploration. Finally, given that Bio‐Oss is prone to displacement and pose operational challenges, there is a pressing need to develop appropriate scaffolds for loading Neo‐treated hDPSCs to boost their clinical applicability.

**Figure 7:**
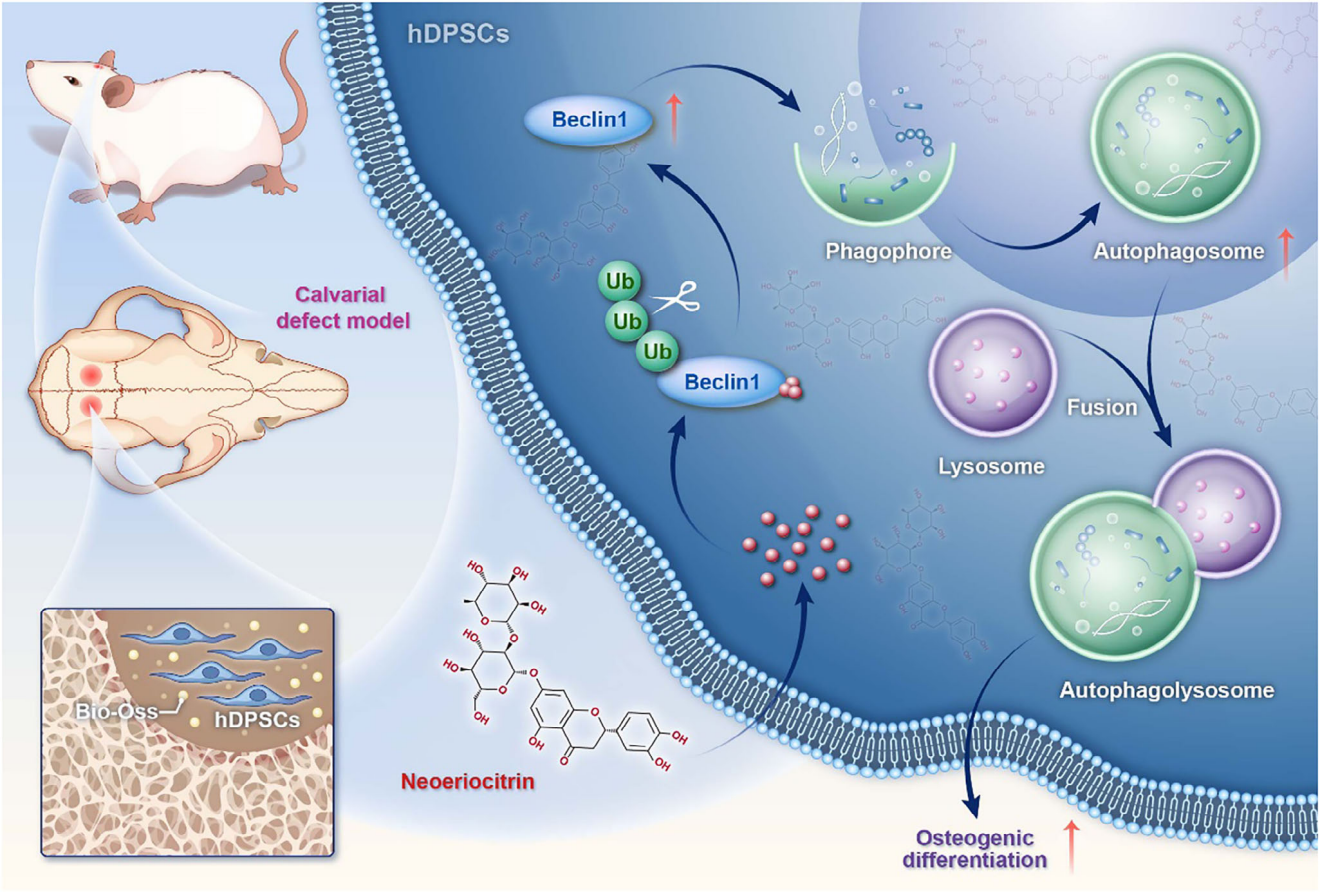
The mechanism diagram of Neo facilitating the osteogenic differentiation of hDPSCs and thereby promoting bone regeneration. Drew by Figdraw.

Overall, this study represents an inaugural demonstration of the impact of Neo on the osteogenic differentiation of hDPSCs and bone regeneration by directly targeting Beclin1 to inhibit its ubiquitination, thereby increasing Beclin1 stability and protein abundance to activate autophagy (**Figure** **7**). This finding provides theoretical support for considering Neo as a potential therapeutic option to enhance bone repair. It also paves the way for developing natural autophagy agonists acting through post‐translational stabilization.

## Conflict of Interest

The authors declare no conflicts of interest.

## Author Contributions

Y.W. performed methodology, software, validation, formal analysis, investigation, wrote the original draft and edited the final manuscript, visualization. H.L. performed methodology, validation, investigation. Q.W. performed methodology, investigation. T.Z. performed methodology, investigation, revised the original draft. R.C. performed methodology, validation. Q.Y. performed methodology, validation. X.T. performed methodology, validation. W.Y. performed conceptualization, revised the original draft. Y.X. performed conceptualization, supervision, revised the original draft. F.Y. performed conceptualization, resources, revised the original draft, supervision, project administration, funding acquisition.

## Supporting information



Supporting Information

Supporting Information

## Data Availability

The data that support the findings of this study are available from the corresponding author upon reasonable request.
