# Supplementary material for: Neoeriocitrin Targeting Beclin1 Deubiquitination and Autophagy in Osteogenic Differentiation of Human Dental Pulp Stem Cells
Source: Adv Sci (Weinh). 2025 Aug 19;12(43):e04378. doi: 10.1002/advs.202504378 (PMC12631872; doi:10.1002/advs.202504378)
Supplement: Supplementary file 1 — Supporting Information [file ADVS-12-e04378-s001.docx]

1. **Supplementary Figures**

**
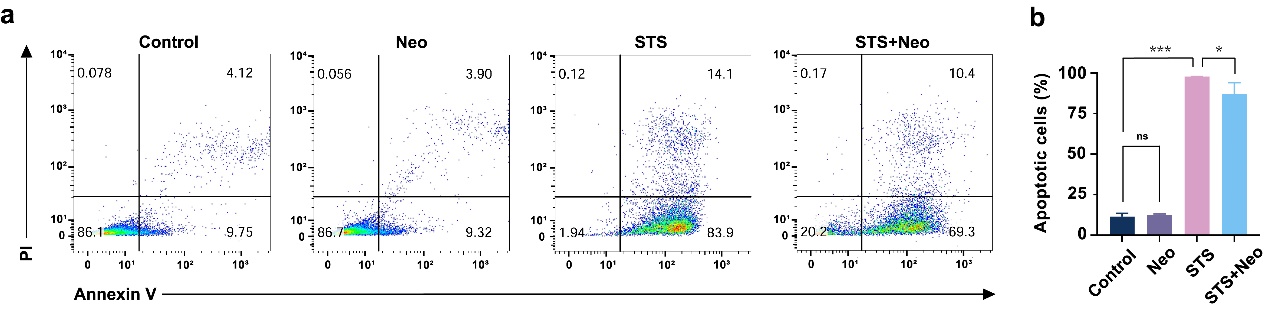
**

**Fig. S1** **Neo does not induce apoptosis and mitigates STS-induced apoptosis in hDPSCs.** (a, b) Flow cytometric analysis of hDPSCs apoptosis after co-culture with various treatments.

**
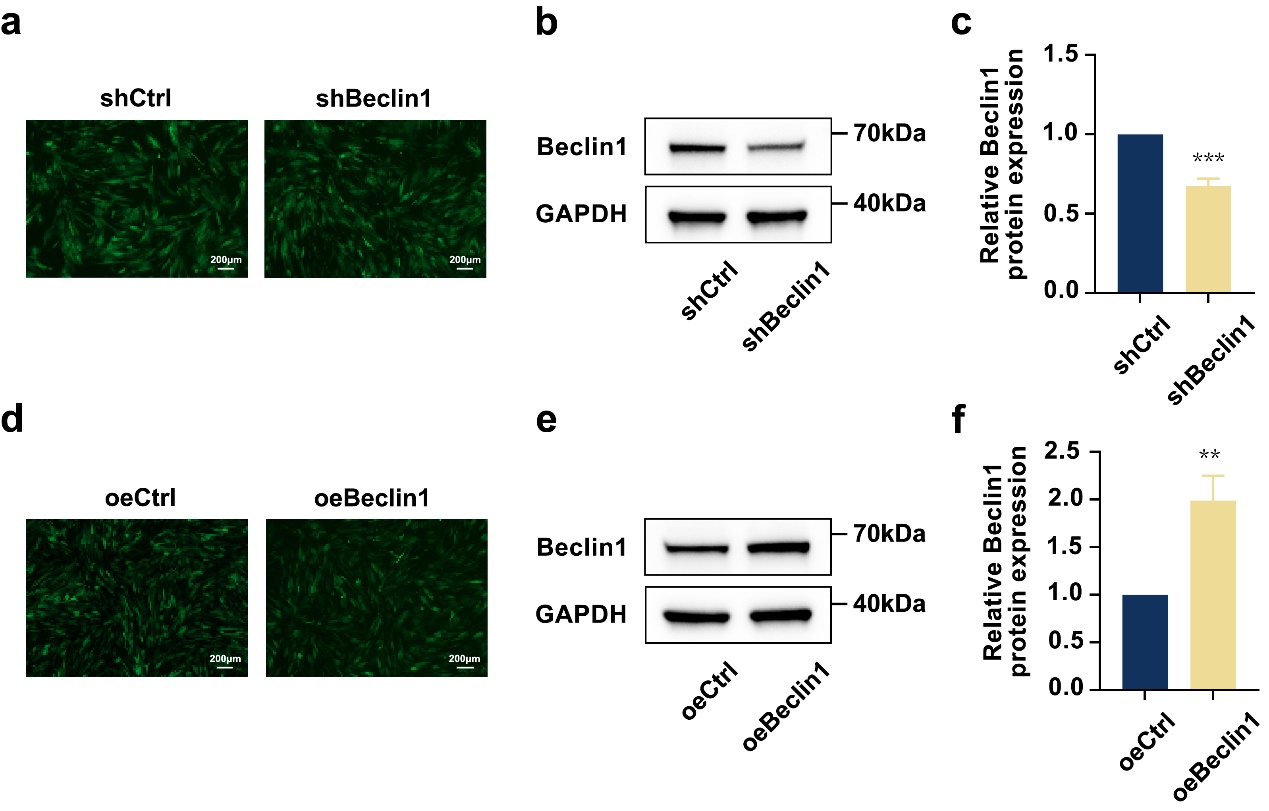
**

**Fig. S2** **The verification of Beclin1 knockdown and overexpression efficiency.** (a-c) Fluorescence microscopy images of hDPSCs transfected with lentiviruses, and corresponding protein expression levels of Beclin1, displaying the efficiency of Beclin1 knockdown (Scale bar: 200 μm). (d-f) Fluorescence microscopy images of hDPSCs transfected with lentiviruses, and corresponding protein expression levels of Beclin1, displaying the efficiency of Beclin1 overexpression (Scale bar: 200 μm). Data expressed as mean ± SD. *: *p* < 0.05, **: *p* < 0.01, ***: *p* < 0.001.


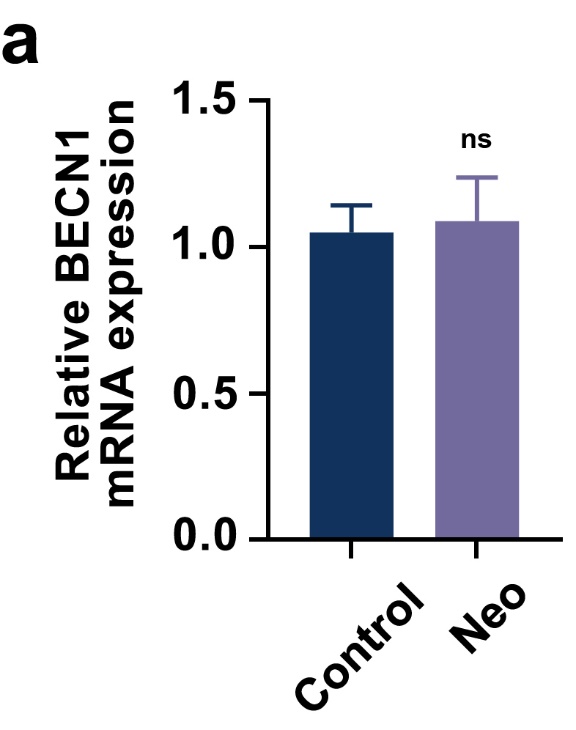


**Fig. S3** **Neo treatment did not alter *BECN1* mRNA levels.** (a) Alterations in mRNA expression levels of *BECN1* measured by qPCR in cells treated with or without Neo. Data expressed as mean ± SD. ns: no significance.

**
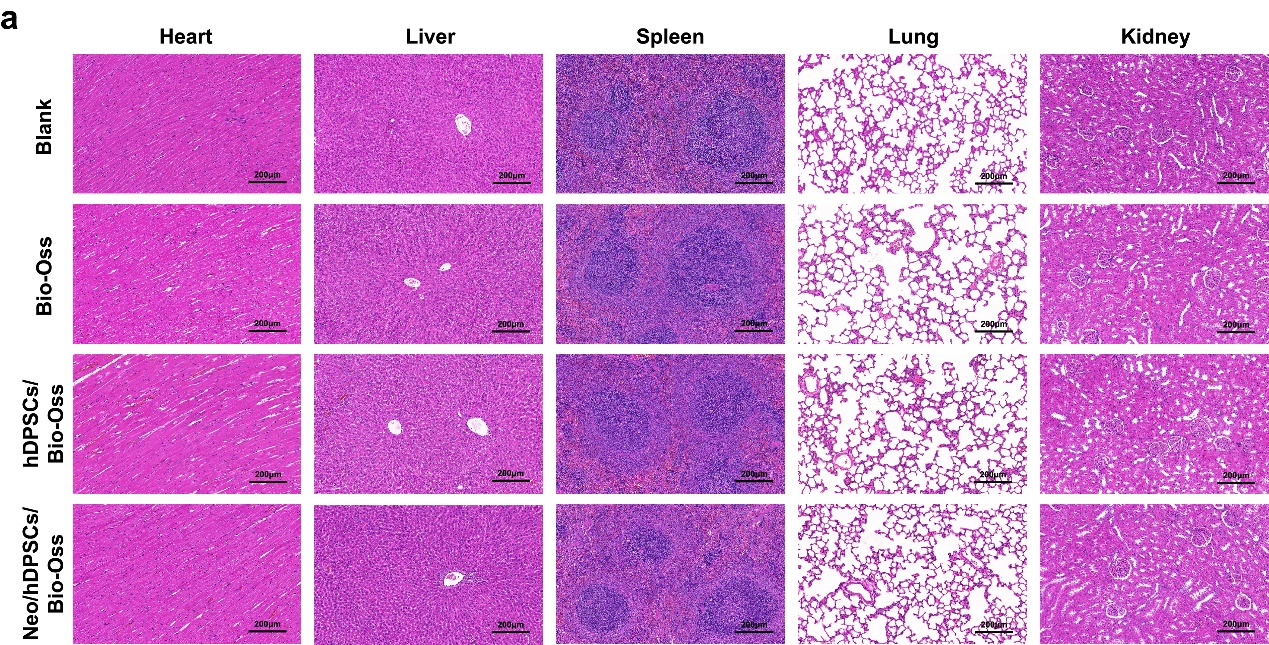
**

**Fig. S4 The biosafety assessment of Neo-stimulated hDPSCs transplantation in the treatment of bone defects.** (a) Representative HE staining images of the hearts, livers, spleens, lungs and kidneys from various groups of rats (Scale bar: 200 μm).

**2. Supplementary Tables**

**Table S1** Primer sequences

| Gene | Primer sequence |
| --- | --- |
| Collagen I | Forward: 5’-GAGGGCCAAGACGAAGACATC-3’  Reverse: 5’-CAGATCACGTCATCGCACAAC-3’ |
| OPN | Forward: 5’-CTCCATTGACTCGAACGACTC-3’  Reverse: 5’-CAGGTCTGCGAAACTTCTTAGAT-3’ |
| ALP | Forward: 5’-ACCTGAGTGCCAGAGTGA-3’  Reverse: 5’-CTTCCTCCTTGTTGGGTT-3’ |
| Runx2 | Forward: 5’-CACTGGCGCTGCAACAAGA-3’  Reverse: 5’-CATTCCGGAGCTCAGCAGAATAA-3’ |
| Beclin1  GAPDH | Forward: 5’-ATCTCGAGAAGGTCCAGGCT-3’  Reverse: 5’-TCTGGGCATAACGCATCTGG-3’  Forward: 5’-GGAGCGAGATCCCTCCAAAAT-3’  Reverse: 5’-GGCTGTTGTCATACTTCTCATGG-3’ |

**Table S2** Statistical results of TPP-Based identification

| Spectrum | | Peptides | | Proteins | |
| --- | --- | --- | --- | --- | --- |
| Total spectrum | Matched spectrum | Peptides | Unique peptides | Identified proteins | Quantified proteins |
| 674629 | 142955 | 48252 | 43920 | 6585 | 4858 |

**Table S3** The mass spectrometric data of TPP

See Excel file.

**Table S4** Blood-routine parameters of rats with different treatments

|  | Blank | Bio-Oss | hDPSCs/Bio-Oss | Neo/hDPSCs/Bio-Oss |
| --- | --- | --- | --- | --- |
| WBC (10^9^/L) | 6.610 ± 1.052 | 6.970 ± 0.539 | 6.713 ± 0.136 | 6.970 ± 0.752 |
| RBC (10^12^/L) | 6.853 ± 0.507 | 7.260 ± 0.086 | 6.920 ± 0.136 | 7.163 ± 0.241 |
| HGB (g/L) | 142.000 ± 3.559 | 143.000 ± 2.160 | 141.333 ± 2.625 | 142.667 ± 2.625 |
| MCV (fL) | 57.233 ± 0.754 | 57.433 ± 0.613 | 57.700 ± 0.374 | 57.500 ± 0.779 |
| PLT (10^9^/L) | 623.667 ± 39.685 | 615.667 ± 58.597 | 655.667 ± 14.704 | 627.000 ± 51.923 |
| LYC# (10^9^/L) | 5.930 ± 0.828 | 5.993 ± 0.201 | 5.877 ± 0.414 | 6.177 ± 0.535 |
| MID# (10^9^/L) | 0.160 ± 0.029 | 0.150 ± 0.050 | 0.130 ± 0.022 | 0.140 ± 0.016 |
| GR# (10^9^/L) | 0.617 ± 0.123 | 0.697 ± 0.150 | 0.497 ± 0.088 | 0.437 ± 0.025 |
| HCT (%) | 39.200 ± 2.861 | 41.733 ± 0.573 | 40.933 ± 0.287 | 41.433 ± 1.053 |
| RDW-CV (%) | 12.233 ± 0.478 | 12.200 ± 0.245 | 12.567 ± 0.170 | 12.400 ± 0.432 |
| MCH (pg) | 19.400 ± 0.216 | 19.700 ± 0.294 | 19.633 ± 0.287 | 19.900 ± 0.356 |

WBC: White blood cell count; RBC: Red blood cell count; HGB: Hemoglobin; MCV: Mean corpuscular volume; PLT: Platelet count; LYC#: Lymphocyte count; MID#: Intermediate cell count; GR#: Granulocyte count; HCT: Hematocrit; RDW: Red blood cell volume distribution width; CV: Coefficient of variation; MCH: Mean corpusular hemoglobin. Data expressed as mean ± SD.

**Table S5** Biochemical indicators of rats with different treatments

|  | Blank | Bio-Oss | hDPSCs/Bio-Oss | Neo/hDPSCs/Bio-Oss |
| --- | --- | --- | --- | --- |
| ALT (U/L) | 40.967 ± 8.029 | 37.900 ± 6.960 | 37.300 ± 3.884 | 38.067 ± 2.738 |
| AST (U/L) | 101.900 ± 17.613 | 82.733 ± 9.151 | 96.367 ± 11.594 | 91.200 ± 6.375 |
| TBIL (μmol/L) | 3.267 ± 0.340 | 3.067 ± 0.047 | 3.233 ± 0.287 | 2.833 ± 0.249 |
| ALB (g/L) | 35.600 ± 6.615 | 30.267 ± 2.538 | 40.267 ± 2.304 | 37.200 ± 2.355 |
| CREA (μmol/L) | 35.633 ± 2.904 | 31.033 ± 3.391 | 38.900 ± 2.286 | 34.200 ±2.328 |
| UREA (mmol/L) | 7.263 ± 0.666 | 7.487 ± 0.433 | 8.027 ± 0.154 | 7.620 ± 0.630 |
| UA (μmol/L) | 35.000 ± 8.042 | 36.000 ± 10.231 | 39.000 ± 11.045 | 41.333 ± 6.799 |
| GLU (mmol/L) | 21.230 ± 2.679 | 17.053 ± 1.022 | 22.013 ± 1.289 | 19.583 ± 0.999 |

ALT: Alanine aminotransferase; AST: Aspartate aminotransferase; TBIL: Total bilirubin; ALB: Albumin; CREA: Creatinine; UREA: Urea, Carbamide; UA: Ursolic acid; GLU: Glucose. Data expressed as mean ± SD.
